# The detection of apical radiolucencies in periapical radiographs: A comparison between an artificial intelligence platform and expert endodontists with CBCT serving as the diagnostic benchmark

**DOI:** 10.1111/iej.14250

**Published:** 2025-05-03

**Authors:** Marwa Allihaibi, Garrit Koller, Francesco Mannocci

**Affiliations:** ^1^ Department of Endodontics Faculty of Dentistry, Taif University Taif Saudi Arabia; ^2^ Department of Endodontics Centre for Oral, Clinical and Translational Sciences, Faculty of Dentistry, Oral & Craniofacial Sciences, King's College London London UK

**Keywords:** artificial intelligence, cone beam computed tomography, endodontics, periapical periodontitis, radiography, dental, sensitivity and specificity

## Abstract

**Aim:**

Accurate detection of periapical radiolucent lesions (PARLs) is crucial for endodontic diagnosis. While cone beam computed tomography (CBCT) is considered the radiographic gold standard for detecting PARLs in non‐root filled teeth, its use is often limited by cost and radiation exposure. Artificial Intelligence (AI)‐based radiographic analysis has shown the potential to enhance the diagnostic capability of periapical (PA) radiographs, but its accuracy, compared with CBCT, needs further evaluation.

The aim of this paper is to assess the diagnostic accuracy of Diagnocat, a commercial AI‐driven platform in detecting PARLs on PA radiographs of teeth diagnosed with irreversible pulpitis or pulp necrosis and scheduled for primary root canal treatment, using CBCT as the reference standard, and to compare Diagnocat's performance with that of experienced clinicians.

**Methodology:**

This retrospective diagnostic accuracy study analysed 339 teeth (796 roots). PA radiographs were independently assessed by two experienced, calibrated endodontists and by Diagnocat. CBCT scans, serving as the reference standard, were evaluated by two different endodontists, blinded to the PA radiograph results. Sensitivity, specificity, accuracy and area under the receiver‐operating characteristic curve (AUC‐ROC) were calculated for Diagnocat and clinicians at both tooth and root levels.

**Results:**

CBCT identified PARLs in 121 (35.7%) teeth and 240 (30.2%) roots. Diagnocat displayed a high level of correlation with clinicians in determining lesion status, with an agreement of 89%. Clinicians demonstrated significantly higher accuracy at the tooth level (86.1% vs. 78.5%, *p* < .001) and greater sensitivity (65.3% vs. 47.9%, *p* < .001) than Diagnocat, while specificity was comparable (97.7% vs. 95.4%, *p* = .3). Similar trends were observed at the root level. AUC‐ROC values were higher for clinicians than Diagnocat at both tooth (0.81 vs. 0.72) and root (0.77 vs. 0.68) levels, although these differences were not statistically significant.

**Conclusion:**

While Diagnocat exhibited high agreement with clinicians in detecting PARLs on PA radiographs, clinicians demonstrated superior accuracy and sensitivity overall. Notably, Diagnocat performed comparably to experienced clinicians in cases without PARLs, highlighting its potential utility for reliably ruling out disease. However, further refinement is required before it can reliably complement clinical judgment in endodontic practice.

## INTRODUCTION

Apical periodontitis (AP) is a chronic inflammatory condition characterized by the destruction of periapical tissues due to the host's immune response to microbial infection in the root canal system (Nair, 2004). This prevalent oral disease affects an estimated 52% of individuals and 5% of teeth globally, posing a significant health burden (Tibúrcio‐Machado et al., 2021). Given its prevalence and impact, accurate diagnosis of AP is crucial in dental practice. It usually includes a combination of clinical evaluation and radiographic assessment, with the latter playing a critical role (Horner et al., 2018; Patel et al., 2009).

Two‐dimensional (2D) periapical (PA) radiographs have been widely used for identifying periapical radiolucent lesions (PARLs) associated with AP. However, whether film‐based or digital, these images have inherent limitations that may hinder accurate diagnosis (Patel et al., 2009). These include the two‐dimensional nature of the images (Azarpazhooh et al., 2016; Webber & Messura, 1999), anatomical noise masking the area of interest (Bender & Seltzer, 2003a; Cotton et al., 2007) and geometric distortion (Forsberg & Halse, 1994; Vande Voorde & Bjorndahl, 1969).

Cone beam computed tomography (CBCT) has been introduced in endodontics, providing three‐dimensional images with higher accuracy, reduced noise and minimal distortion (Scarfe & Farman, 2008), effectively addressing many of the shortcomings of conventional radiographic techniques. The superiority of CBCT in detecting PARLs has been consistently demonstrated in various studies. For instance, the prevalence of PARLs detected by CBCT ranges from 5.8% (Dutta et al., 2014) to 10.4% (Meirinhos et al., 2020) of examined teeth, and it is significantly higher than that recorded using PA radiographs. Moreover, CBCT has been validated as the radiographic gold standard for AP diagnosis through histopathological studies on human cadavers. Kanagasingam et al. (2017) demonstrated that CBCTs had significantly higher sensitivity (0.89,) in detecting AP compared with periapical radiography (0.38) using histology as the gold standard. These findings were further supported by another histologic study by Kruse et al. (2019), finding that CBCT had similar sensitivity (0.95) in identifying mild AP in treatment‐naïve teeth.

Despite its superior accuracy, CBCT has limitations, including higher costs, increased radiation exposure and limited availability (Patel et al., 2019), leading to the continued use of 2D radiographs. This highlights the need for improved diagnostic accuracy within the scope of 2D imaging modalities.

In recent years, artificial intelligence (AI) has emerged as a promising tool to enhance diagnostic accuracy in various medical fields, including dentistry, with AI‐based tools utilizing deep learning and convolutional neural networks showing promising results in analysing radiographic images (Khanagar et al., 2021) and detecting periapical radiolucent lesions (Sadr et al., 2023).

These advances have led to the development of several AI‐driven diagnostic platforms in dentistry. Commercially available AI‐driven platforms, such as Diagnocat (Diagnocat San Francisco, CA, USA), utilize neural networks trained on large datasets to provide dental diagnostics (Dvoyris, 2023). The system utilizes convolutional neural networks to analyse periapical radiographs and identify potential pathologies, including apical radiolucencies. The system is intended to assist clinicians in diagnosis and treatment planning by serving as a screening and decision‐support tool. While a recent study evaluating Diagnocat demonstrated promising results in periapical radiograph analysis (Issa et al., 2023), the study had limitations. It was based on a small sample size and relied on ground‐truth assessments from a single oral and maxillofacial radiology expert and one trainee rather than employing CBCT as the reference standard.

Given these prior limitations and the need for further validation, the aim of this study was to assess the performance of Diagnocat in detecting apical radiolucencies on periapical radiographs of teeth without prior root canal treatment, using CBCT as the reference standard. Specifically, our objectives were to:

1. Determine the sensitivity, specificity and overall accuracy of Diagnocat in detecting apical radiolucencies on periapical radiographs of untreated teeth compared with CBCT findings.
2. Compare the apical radiolucency detection performance of Diagnocat against that of experienced clinicians on periapical radiographs of untreated teeth, using CBCT as the reference standard.

We hypothesize that there may be differences in the diagnostic performance between the Diagnocat and experienced clinicians when using CBCT as the reference standard.

## MATERIALS AND METHODS

### Study design and ethical approval

This retrospective diagnostic accuracy study was conducted following the Standards for Reporting Diagnostic Accuracy Studies (STARD) 2015 (Bossuyt et al., 2015) and the Preferred Reporting Items for Diagnostic Accuracy Studies in Endodontics (PRIDASE) 2024 guidelines (Nagendrababu et al., 2024). The study analysed the periapical status of 339 teeth from 334 preoperative periapical radiographs and their corresponding CBCT images from four prospective clinical outcome studies (Knight et al., 2020; Patel et al., 2025; Rahim, 2024; Zahran et al., 2021) conducted at Guy's and St Thomas' NHS Foundation Trust (London, UK). The current study was designed and executed after the completion of these original trials, using existing radiographic data. The reference standard CBCT‐based imaging series (ground truth) were performed during the original prospective studies (Knight et al., 2020; Patel et al., 2025; Rahim, 2024; Zahran et al., 2021) before the design of this retrospective analysis. The CBCT scans were obtained as part of different research protocols, independent of any clinical indications.

Given the retrospective, fully anonymized nature of the data, this study was deemed a non‐interventional clinical trial under a standardized non‐experimental protocol. Approval for conducting this analysis was obtained from the GERRI Oversight Committee (Rec Reference: 20/EM/0112).

As part of the original prospective trials (Knight et al., 2020; Patel et al., 2025; Rahim, 2024; Zahran et al., 2021), all patients provided informed written consent for their anonymized data to be used for research purposes. Patients were informed about voluntary participation, confidentiality and the right to withdraw without affecting treatment. Consent forms were securely stored in locked cabinets accessible only to investigators.

### Participant selection

- *Inclusion criteria*: Teeth indicated for primary root canal treatment with a confirmed diagnosis of either irreversible pulpitis or necrotic pulp, and both periapical radiographs and CBCT scans are available.
- *Exclusion criteria*: Missing or incomplete radiographic data (including cases with inadequate image quality preventing definitive assessment).
- *Participant selection*: Cases were collected from four prospective clinical trials (Knight et al., 2020; Patel et al., 2025; Rahim, 2024; Zahran et al., 2021) conducted at Guy's and St Thomas' NHS Foundation Trust (London, UK), between 2012 and 2022. A convenience sample was used, including all eligible cases.

### Radiographic assessment

CBCT images from the previously mentioned trials had been assessed by two experienced endodontists following the protocol described in previous research by Patel et al. (2012). Briefly, the evaluation began with identifying CBCT images that best confirmed the presence or absence of radiolucent periapical lesions attached to the root apex in sagittal, coronal and/or axial planes. The evaluators also had access to the full CBCT dataset, enabling them to scroll through slices without additional multiplanar reconstructions. This allowed for the assessment of lesions directly associated with the root apex, ensuring a focused and standardized approach while also enabling evaluators to rule out non‐apical lesions or incidental findings.

The PA radiographs were independently evaluated by two different experienced endodontists, with the brightness and contrast of all images adjusted to enhance the visualization of periapical radiolucencies. No cropping or further manipulation of the images was performed. Assessments were conducted blinded to clinical information, as well as Diagnocat results and CBCT findings.

The evaluation process included radiographic scoring, viewing sessions, examiner calibration and intra‐examiner reliability testing. Intra‐examiner Kappa scores were 0.90 for CBCT and 0.81 for PA, while inter‐examiner agreement was 0.71 for CBCT and 0.73 for PA.

The diagnostic cut‐off for diagnosing a periapical lesion by the clinicians on both PA radiographs and CBCT images was set at a radiolucency measuring at least twice the width of the periodontal ligament space, as defined by Bornstein et al. (2011) and Low et al. (2008). For multi‐rooted teeth, a lesion was identified if it was detected in at least one of the roots.

PA radiographs were taken using a standardized paralleling technique using a dental X‐ray machine (Planmeca Prostyle Intra, Helsinki, Finland).

### AI‐driven platform (Diagnocat) description and protocol

The AI‐driven platform (Diagnocat, USA) was utilized for the automated analysis of anonymized digital periapical radiographs. In this study, the platform's default setting, which highlights lesions with a probability higher than 50% in the automatically generated report, was utilized. Diagnocat automatically detected and highlighted apical radiolucencies on the radiographs without requiring any specific enhancement, cropping or human intervention. When a highlighted region involved multiple roots, all affected roots were recorded as exhibiting apical radiolucencies. Diagnocat's fully automated analysis eliminated the need for operator experience or calibration. Diagnocat results were recorded independently and compared with assessments from human examiners and the reference standard to evaluate the accuracy of Diagnocat. Additional analyses were performed evaluating Diagnocat's low probability detection feature (higher than 30%) to assess its impact on diagnostic performance (refer to Figure S1 and Tables S5–S10 for detailed results).

### Reference standard (CBCT assessment)

Small‐field CBCT scans (4 × 4 cm) were acquired using a 3D Accuitomo CBCT scanner (J. Morita, Kyoto, Japan) as part of the original trial protocols.

### Management of missing and indeterminate data

To ensure data integrity, 76 cases were excluded from the eligible dataset: 69 due to the absence of either PA or CBCT radiographs and 7 due to poor image quality that hindered definitive assessment. No data imputation was undertaken; only cases with complete and evaluable data were included in the final analysis.

### Statistical analysis

Data were analysed using SPSS version 29 (IBM Armonk, NY, USA). Key diagnostic accuracy measures, including sensitivity, specificity and accuracy, were calculated using CBCT as the reference standard for both Diagnocat and clinicians. For these diagnostic accuracy metrics, 95% confidence intervals were calculated using Wilson score intervals for larger samples (*n* ≥ 60) and exact (Clopper‐Pearson) intervals for smaller samples (*n* < 30). Diagnostic accuracy between Diagnocat and clinicians was assessed using McNemar's test. Inter‐rater agreement was assessed using Cohen's kappa coefficient.

Receiver‐operating characteristic (ROC) curves were plotted to compare Diagnocat and clinician performance in detecting apical radiolucencies on periapical radiographs. The area under the curve (AUC) was calculated to quantify diagnostic performance, with DeLong's test used to compare AUCs between Diagnocat and clinicians (*p* < .05 considered significant).

Exploratory subgroup analyses examined variability in diagnostic accuracy based on tooth type and root canal system complexity. These analyses were not pre‐specified in the original protocol.

Due to the retrospective design of this analysis, an a priori sample size calculation was not conducted. Instead, a post‐hoc power analysis was carried out using G*Power 3.1.9.6 software (Faul et al., 2007). This analysis indicated large effect sizes (OR = 9.5 for proportion 0.10 of discordant pairs in accuracy of AI vs. clinicians) at both the tooth level (*N* = 339) and root level (*N* = 796), achieving a statistical power of 0.99 using McNemar's test. These results confirm that the sample size was sufficient to detect significant differences in diagnostic accuracy between Diagnocat and clinician assessments at both analytical levels.

## RESULTS

### Sample characteristics

**FIGURE 1:**
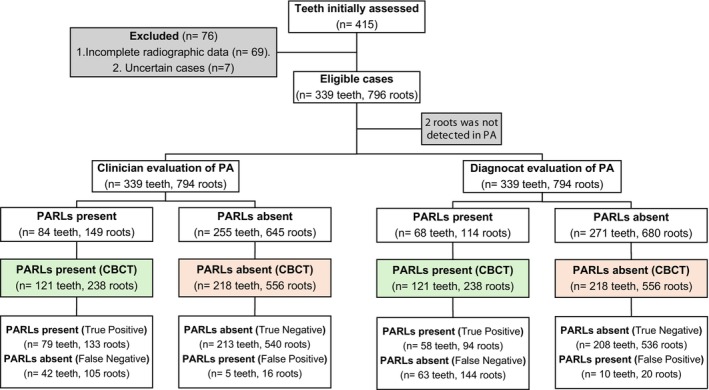
Flow diagram showing the selection and analysis of teeth and roots in the study, following the STARD 2015 guidelines. CBCT, cone beam computed tomography; PA, periapical radiograph; PARLs, periapical radiolucencies. DeLong's test (*p*‐value: .88). DeLong's test (*p*‐value: .87).

The flow of participants through the study is presented in Figure 1, following the STARD 2015 guidelines (Bossuyt et al., 2015). A total of 339 teeth (137 [40.4%] maxillary; 202 [59.6%] mandibular) and 796 roots (394 [49.5%] maxillary; 402 [50.5%] mandibular) were included. The sample predominantly consisted of molars (327 teeth [96.5%]; 782 roots [98.2%]), with a smaller number of anterior teeth and premolars. CBCT identified PARLs in 121 (35.7%) teeth and 240 (30.2%) roots. Notably, 2 (0.3%) roots were visible only on CBCT and undetectable on PA radiographs.

### Cross‐tabulation of index test and reference standard results

**FIGURE 2:**
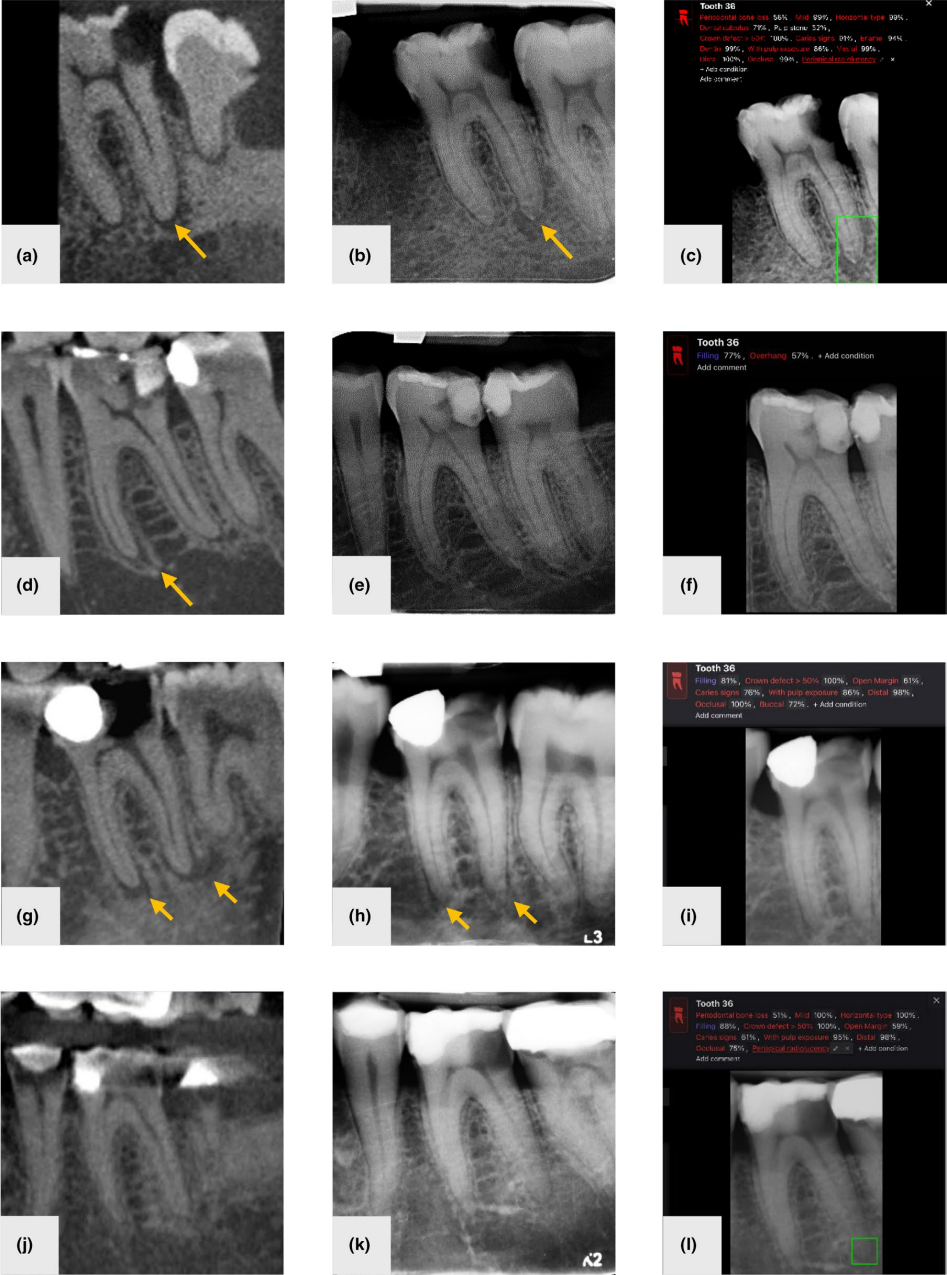
Comparison of periapical radiolucencies detection between CBCT ground truth, clinician assessment of periapical radiographs and Diagnocat detection by Diagnocat. (a–c) True positive cases: (a) CBCT slice with arrow indicating distal root lesion, (b) radiograph with arrow indicating distal root lesion, (c) Diagnocat output with green box around distal root lesion. (d–f) False negative cases(Diagnocat and clinicians): (d) CBCT slice with arrow indicating mesial root lesion, (e) radiograph, (f) Diagnocat output. (g–i) False negative cases by Diagnocat: (g) CBCT slice with arrows indicating mesial and distal root lesions, (h) radiograph with arrows indicating mesial and distal root lesions, (i) Diagnocat output. (j–l) False positive cases by Diagnocat: (j) CBCT slice, (k) radiograph, (l) Diagnocat output with green box around distal root.

The cross‐tabulation of index test results by the reference standard for clinicians and Diagnocat at both tooth and root levels is presented in Tables S1 and S2, respectively. At tooth level, clinicians demonstrated a true positive rate of 65.3% (79/121) and a true negative rate of 97.7% (213/218) for teeth with and without PARLs, respectively. In comparison, Diagnocat yielded a true positive rate of 47.9% (58/121) and a true negative rate of 95.4% (208/218). A similar pattern was observed at root level. Representative cases illustrating true positive, false negative and false positive results are shown in Figure 2.

### Agreement between Diagnocat and clinicians in periapical lesion detection

Overall, agreement between clinicians and Diagnocat in detecting PARLs was high at both tooth level (88.8%; Cohen's *κ* = 0.68) and root level (89.5%; *κ* = 0.62). Agreement was higher for PARL‐negative cases than for PARL‐positive cases at both levels. Notably, in PARL‐positive cases, clinicians and Diagnocat jointly missed 33.9% of PARLs at the tooth level and 40.3% at the root level. For PARL‐negative cases, both agreed on 93.1% of teeth and 95.3% of roots, with joint overdiagnosis occurring in less than 1% of PARL‐negative roots.

### Combined Diagnocat and clinician performance in PARLs detection

Of the 339 teeth, Diagnocat and clinicians together correctly classified 260 teeth (76.7%) based on the gold standard. For the remaining cases, clinicians correctly classified an additional 32 teeth (9.4%), while Diagnocat correctly classified 6 teeth (1.8%), resulting in a total of 298 teeth (87.9%) correctly classified when combining both methods (Table S3).

Similarly, out of 794 roots, Diagnocat and clinicians jointly classified 610 roots (76.8%) correctly. Clinicians alone classified an additional 63 roots (7.9%), while Diagnocat classified 20 roots (2.5%), leading to a total of 693 roots (87.3%) correctly classified when their results were combined (Table S4).

In terms of missed diagnoses, both Diagnocat and clinicians failed to detect lesions in 41 teeth (12.1%) and 101 roots (12.7%).

### Diagnostic accuracy metrics

Tables 1 and 2 show the diagnostic accuracy metrics at the tooth and root levels, respectively. Clinicians consistently demonstrated higher overall accuracy (teeth: 86.1% vs. 78.5%, *p* < .001; roots: 84.8% vs. 79.4%, *p* < .001) and sensitivity (teeth: 65.3% vs. 47.9%, *p* < .001; roots: 55.9% vs. 39.5%, *p* < .001) compared to Diagnocat, with comparable specificity.

**TABLE 1:** Diagnostic performance of clinicians and diagnocat in detecting periapical radiolucencies: tooth‐level analysis of sensitivity, specificity and accuracy by dental arch and tooth type.

(*N*, %)	Sensitivity			Specificity			Accuracy		
	Clinician (95% CI)	Diagnocat (95% CI)	*p*‐Value^a^	Clinician (95% CI)	Diagnocat (95% CI)	*p*‐Value^a^	Clinician (95% CI)	Diagnocat (95% CI)	*p*‐Value^a^
All teeth (339, 100%)	65.3% (56.81, 73.77)	47.9% (39.03, 56.84)	<.001***	97.7% (95.72, 99.69)	95.4% (92.64, 98.19)	.302	86.1% (82.46, 89.81)	78.5% (74.09, 82.84)	<.001***
Arch									
Upper (137, 40.4%)	66.0% (53.29, 78.79)	41.5% (28.24, 54.78)	<.001***	97.6% (94.36, 100)	97.6% (94.36, 100)	1.0	85.4% (79.49, 91.31)	75.9% (68.75, 83.07)	.002**
Lower (202, 59.6%)	64.7% (53.35, 76.06)	52.9% (41.08, 64.8)	.021*	97.8% (95.26, 100)	94.0% (90.02, 98.04)	.227	86.6% (81.94, 91.33)	80.2% (74.7, 85.69)	.007**
Tooth type^b^									
Anterior (10, 2.9%)	100% (67.56, 100)	62.5% (30.57, 86.32)	.25	100% (34.24, 100)	50.0% (9.45, 90.55)	1.0	100% (72.25, 100)	60.0% (31.27, 83.18)	.125
Molar (327, 96.4%)	62.8% (53.92, 71.74)	46.9% (37.70, 56.10)	<.001***	97.7% (95.64, 99.69)	95.8% (93.11, 98.48)	.424	85.6% (81.82, 89.43)	78.9% (74.48, 83.32)	<.001***
Teeth^b^									
L6^c^ (126, 37.2%)	73.3% (60.41, 86.25)	66.7% (52.89, 80.44)	.375	100% (100.0, 100)	93.8% (88.59, 99.07)	.063	90.5% (85.35, 95.6)	84.1% (77.75, 90.51)	.021*
L7^c^ (70, 20.6%)	36.8% (15.15, 58.53)	21.1% (2.72, 39.38)	.25	94.1% (87.66, 100)	96.1% (90.75, 100)	1.0	78.6% (68.96, 88.18)	75.7% (65.67, 85.76)	.727
U6^c^ (79, 23.3%)	65.7% (49.99, 81.44)	40.0% (23.77, 56.23)	.004**	97.7% (93.32, 100)	100% (100.0, 100)	1.0	83.5% (75.37, 91.72)	73.4% (63.68, 83.16)	.021*
U7^c^ (51, 15.0%)	57.1% (31.22, 83.07)	35.7% (10.61, 60.81)	.25	97.3% (92.07, 100)	94.6% (87.31, 100)	1.0	86.3% (76.83, 95.72)	78.4% (67.14, 89.72)	.219

^a^ McNemar test used for clinician‐Diagnocat comparison.

^b^ Teeth with <5 counts excluded.

^c^ Tooth notation: L6 = mandibular first molar; L7 = mandibular second molar; U6 = maxillary first molar; U7 = maxillary second molar.

*p < .05, **p < .01, ***p < .001.

**TABLE 2:** Diagnostic performance of clinicians and diagnocat in detecting periapical radiolucencies: root‐level analysis of sensitivity, specificity and accuracy by dental arch, tooth type and root type.

(*N*, %)	Sensitivity			Specificity			Accuracy		
	Clinician (95% CI)	Diagnocat (95% CI)	*p*‐Value^a^	Clinician (95% CI)	Diagnocat (95% CI)	*p*‐Value^a^	Clinician (95% CI)	Diagnocat (95% CI)	*p*‐Value^a^
All roots (794, 100%)	55.9% (49.57, 62.19)	39.5% (33.29, 45.71)	<.001***	97.1% (95.73, 98.51)	96.4% (94.85, 97.95)	.556	84.8% (82.26, 87.26)	79.4% (76.53, 82.16)	<.001***
Arch									
Upper (394, 49.6%)	50.4% (41.7, 59.09)	31.5% (23.42, 39.57)	<.001***	96.6% (94.46, 98.79)	97.0% (94.96, 99.05)	1.0	81.7% (77.91, 85.54)	75.9% (71.66, 80.11)	.001***
Lower (400, 50.4%)	62.2% (53.14, 71.18)	48.7% (39.35, 57.95)	.001**	97.6% (95.81, 99.35)	95.9% (93.55, 98.15)	.302	87.8% (84.54, 90.96)	82.8% (79.05, 86.45)	.001***
Tooth type^b^									
Anterior (10, 1.3%)	100% (67.56, 100)	62.5% (30.57, 86.32)	.25	100% (34.24, 100)	50.0% (9.45, 90.55)	1.0	100% (72.25, 100)	60.0% (31.27, 83.18)	.125
Molar (782, 98.5%)	54.4% (47.91, 60.79)	38.7% (32.40, 44.99)	<.001***	97.1% (95.7, 98.5)	96.6% (95.04, 98.08)	.69	84.5% (81.99, 87.06)	79.5% (76.71, 82.37)	<.001***
Root^b^									
Distal (196, 24.7%)	54.7% (41.32, 68.12)	47.2% (33.73, 60.61)	.344	97.9% (95.55, 100)	96.5% (93.49, 99.51)	.687	86.2% (81.4, 91.05)	83.2% (77.92, 88.4)	.21
Distobuccal (128, 16.1%)	46.2% (30.51, 61.8)	20.5% (7.84, 33.19)	.006*	94.4% (89.6, 99.17)	96.6% (92.88, 100)	.625	79.7% (72.72, 86.66)	73.44% (65.79, 81.09)	.077
Mesial (196, 24.7%)	66.7% (53.73, 79.6)	51.0% (37.26, 64.7)	.008**	97.2% (94.58, 99.91)	95.9% (92.62, 99.1)	.727	89.3% (84.96, 93.62)	84.2% (79.08, 89.29)	.021*
Mesiobuccal (128, 16.1%)	55.0% (39.58, 70.42)	37.5% (22.5, 52.5)	.065	97.7% (94.61, 100)	97.7% (94.61, 100)	1.0	84.4% (78.08, 90.67)	78.9% (71.84, 85.97)	.118
Palatal (129, 16.3%)	45.2% (30.19, 60.29)	31.0% (16.97, 44.93)	.146	97.6% (94.55, 100)	96.6% (92.72, 100)	1.0	80.6% (73.8, 87.44)	75.2% (67.74, 82.65)	.118
Single rooted (14, 1.8%)	90.0% (71.41, 100)	60% (29.64, 90.36)	.25	100.0% (100.0, 100)	75% (32.56, 100)	1.0	92.9% (79.37, 100)	64.3% (39.19, 89.39)	.125

^a^ McNemar test used for clinician Diagnocat comparison.

^b^ Roots with <5 counts excluded.

*p < .05, **p < .01, ***p < .001.

### ROC analysis

**FIGURE 3:**
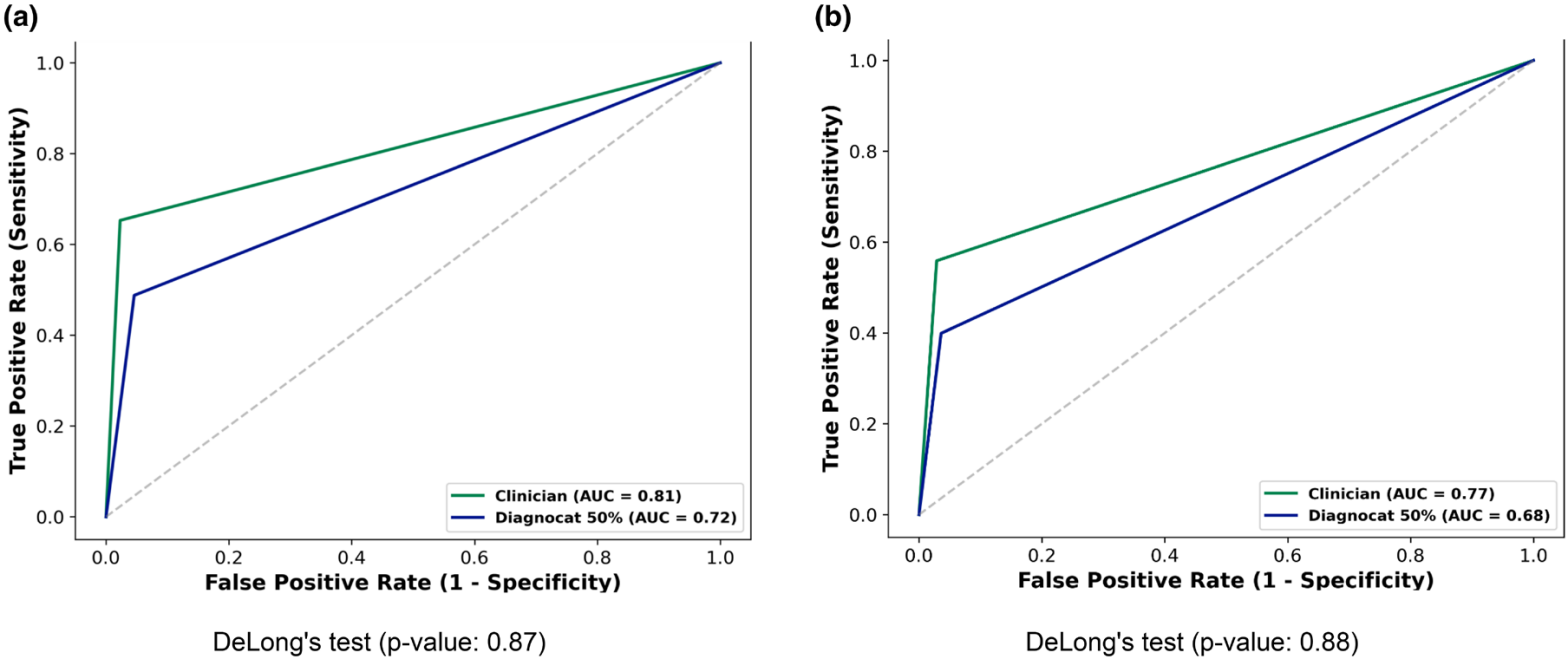
Receiver‐operating characteristic (ROC) curves comparing Diagnocat with probability higher than 50% and clinician performance in detecting apical radiolucencies on periapical radiographs, using cone beam computed tomography (CBCT) as the reference standard, at the (a) tooth level and (b) root level.

ROC curve analysis (Figure 3) showed that clinicians achieved higher areas under the curve (AUC) compared with Diagnocat at both the tooth level (0.81 vs. 0.72) and the root level (0.77 vs. 0.68). However, these differences were insignificant (DeLong's test, *p* > .05).

## DISCUSSION

The present study aimed to compare the diagnostic performance of Diagnocat, an AI‐driven platform, with that of experienced clinicians in detecting PARLs in periapical radiographs. Matching CBCT imaging series were used as the ground‐truth reference standard. Our findings demonstrated a high level of agreement between Diagnocat and clinicians when assessing the presence and absence of PARL on PA radiographs at both the tooth level and root level. These findings align with previous studies, such as Sadr et al. (2023), who reported pooled sensitivity (0.925) and specificity (0.852) for deep learning models. Such performance likely stems from Diagnocat's machine learning foundations, which are trained on expert‐annotated 2D periapical radiographs, enabling Diagnocat to replicate human diagnostic patterns while inheriting similar limitations; however, when performance was evaluated against the CBCT reference standard, clinicians outperformed Diagnocat in both accuracy and sensitivity. This reflects clinicians' superiority in identifying true positives, which is essential for reducing missed diagnoses. Specificity, however, was comparable between the two, indicating similar effectiveness in identifying the absence of lesions. The consistently low sensitivity observed in both groups underscores the inherent challenge of detecting radiolucencies with 2D imaging alone, reinforcing CBCT's role as the gold standard for the radiographic detection of apical radiolucencies in non‐root canal treated teeth.

By contrast, Issa et al. (2023) reported considerably higher performance metrics for Diagnocat, with a sensitivity of 92.30% and specificity of 97.87%. The lower sensitivity observed in our study, particularly at the tooth level (47.9%), likely reflects key methodological differences. Issa et al. relied on clinician assessments and a smaller sample of 60 teeth, while our study used CBCT as the reference standard and a larger sample of 339 teeth.

The performance gap between clinicians and Diagnocat varied across the dental arches, revealing patterns consistent with the anticipated challenges of radiographic interpretation due to anatomical complexity.

In the upper arch, clinicians demonstrated substantially higher sensitivity (teeth: 66.0% vs. 41.5%; roots: 50.4% vs. 31.5%) and accuracy (teeth: 85.4% vs. 75.9%; roots: 81.7% vs. 75.9%) compared with Diagnocat. The complex anatomy of the posterior maxilla, including the shallow palatal vault and divergent root configurations of multi‐rooted teeth, contributes to these discrepancies. These features complicate the positioning of the periapical image receptor, leading to geometric distortion that can either alter the perceived size of radiolucencies or obscure them entirely (Bender et al., 1966; Bender & Seltzer, 2003a; Huumonen & Ørstavik, 2002; Lofthag‐Hansen et al., 2007). Experienced clinicians were more likely to recognize the limitations of 2D imaging and adapt to these distortions through thorough assessment, a skill that current AI systems have yet to develop.

The performance of both clinician and Diagnocat varied across specific molar teeth and their roots. In first upper molars (U6), clinicians demonstrated significantly higher sensitivity (65.7% vs. 40.0%) and accuracy (83.5% vs. 73.4%). While the difference in second upper molars (U7) was not statistically significant, the performance gap remained notable. Both Diagnocat and clinicians exhibited low sensitivity in detecting radiolucencies in upper molars, consistent with Goldman et al. (1972), who observed that the anatomical complexity of maxillary molars often leads to significant examiner disagreement. This phenomenon is attributed to the divergent or convergent root anatomy of multi‐rooted teeth, which makes it nearly impossible to eliminate geometric distortion and magnification entirely, particularly in the posterior maxilla (Lofthag‐Hansen et al., 2007).

In the analysis of specific roots of upper molars, clinicians demonstrated higher diagnostic accuracy of radiolucencies compared with Diagnocat. Within clinicians' assessments, sensitivity was lowest in the palatal root, attributable to unique anatomical challenges. These challenges include proximity to the maxillary sinus floor and superimposition of buccal roots over the palatal root in 2D images, which can obscure critical diagnostic details (Low et al., 2008). Diagnocat also struggled to differentiate these subtle radiographic features, resulting in even lower sensitivity in this region.

The performance gap between clinicians and Diagnocat narrowed in the lower arch, with clinicians still outperforming Diagnocat. This reduced difference may be attributed to the relatively clearer, consistent imaging of lower molars, with fewer anatomical challenges than their upper counterparts in terms of positioning the periapical image receptor and capturing the roots without overlapping. Clinicians showed better sensitivity and accuracy in first lower molars (L6). However, both groups demonstrated the lowest sensitivity in second lower molars (L7), attributable to the posterior position and increased bone density. The denser overlying cortical plate often obscures lesions in this area (Bender & Seltzer, 2003a, 2003b; Schwartz & Foster, 1971). These findings align with studies showing CBCT's superiority over 2D radiographs in detecting mandibular posterior lesions (Bornstein et al., 2011).

While clinicians and the Diagnocat jointly identified correct cases in the majority of teeth and roots, each method demonstrated unique detection capabilities. Clinicians contributed substantially more additional correct identifications than Diagnocat, suggesting their superior diagnostic ability. However, Diagnocat showed the capacity to identify a small but noteworthy proportion of cases missed by clinicians, particularly in challenging anatomical regions. This pattern suggests potential value in using Diagnocat as an adjunctive tool rather than a primary diagnostic method. Nevertheless, the persistence of joint failures in both tooth and root assessment indicates that even a combined approach cannot fully overcome the inherent limitations of 2D radiographic assessment.

However, implementing such a combined approach presents additional challenges. Unlike clinicians who can explain their diagnostic reasoning, the underlying algorithmic processes of AI systems remain inaccessible, making it difficult to understand how they identify cases missed by humans.

Additional analysis of diagnostic thresholds demonstrated that at a lower probability threshold (>30%), Diagnocat showed increased sensitivity (teeth: 70.3%; roots: 55.0%) but with concurrent reductions in specificity (teeth: 78.0%; roots: 86.2%) and accuracy (teeth: 75.2%; roots: 76.8%). Despite this improved detection capability, Diagnocat's performance remained inferior to that of experienced clinicians. The marked decline in specificity raises significant concerns regarding potential overdiagnosis and unnecessary interventions.

The study's strengths include the use of CBCT as a high‐accuracy reference standard, a large sample size focused on complex cases, and evaluations by experienced endodontists under blinded conditions. However, several limitations of this study should be acknowledged. While the sample size is substantial, it may not capture the full range of variations in the presentation of apical radiolucencies, and the predominance of molars, which is associated with the nature of the clinical studies for which the teeth were selected, limits the generalizability of findings to other tooth types. Additionally, the exclusive use of Diagnocat as the sole AI system may limit the applicability of the findings to other AI systems with different algorithms or training datasets. Furthermore, the retrospective design introduces the potential for selection and information biases, despite efforts to minimize these through blinded evaluation and the use of a robust reference standard.

Further prospective research with larger, more balanced samples across all tooth types and possibly involving less experienced operators is essential to determine the generalizability of these findings.

## CONCLUSION

This study compared the diagnostic accuracy of Diagnocat, an AI‐driven platform, with that of experienced clinicians in detecting apical radiolucencies on periapical radiographs, using CBCT as the reference standard. Clinicians outperformed Diagnocat in both accuracy and sensitivity, while specificity was comparable. The high specificity of both suggests that Diagnocat may have utility in ruling out disease, though it should currently be viewed as an adjunctive tool. While Diagnocat shows promise, expert clinicians continue to demonstrate superior diagnostic performance. Further research is required before the AI‐driven platforms can be considered a reliable standalone diagnostic tool in this domain.

## AUTHOR CONTRIBUTIONS

**Marwa Allihaibi:** Conceptualization, methodology, investigation, data curation, validation, visualization, writing—original draft, writing—review and editing, project administration. **Garrit Koller:** Supervision, conceptualization, methodology, formal analysis, validation, visualization, writing—review and editing. **Francesco Mannocci:** Supervision, conceptualization, methodology, formal analysis, validation, visualization, writing—review and editing.

## FUNDING INFORMATION

Marwa Allihaibi is a PhD candidate supported by a scholarship from Taif University, Taif, Saudi Arabia.

## CONFLICT OF INTEREST STATEMENT

The authors declare no conflicts of interest related to this study.

## ETHICS STATEMENT

This study was a retrospective diagnostic accuracy study, following a standardized non‐experimental protocol. Approval was obtained from the Guy's and St Thomas' Hospital Electronic Record Research Interface (GERRI) Oversight Committee (IRAS ID: 257283, REC Reference: 20/EM/0112, extension granted 28/11/2023).

## Supporting information


Data S1

## Data Availability

The data that support the findings of this study are available from the corresponding author upon reasonable request. As this study is retrospective, separate registration was not required. A comprehensive description of the study methods, including the statistical analysis plan, is also available from the corresponding author upon request.

## References

[iej14250-bib-0001] Azarpazhooh, A. , Dao, T. , Ungar, W.J. , Da Costa, J. , Figueiredo, R. , Krahn, M. et al. (2016) Patients' values related to treatment options for teeth with apical periodontitis. Journal of Endodontics, 42, 365–370. 26778269 10.1016/j.joen.2015.11.022

[iej14250-bib-0002] Bender, I.B. & Seltzer, S. (2003a) Roentgenographic and direct observation of experimental lesions in bone: I. 1961. Journal of Endodontics, 29, 702–706; discussion 701. 14651274 10.1097/00004770-200311000-00005

[iej14250-bib-0003] Bender, I.B. & Seltzer, S. (2003b) Roentgenographic and direct observation of experimental lesions in bone: II. 1961. Journal of Endodontics, 29, 707–712; discussion 701. 14651275 10.1097/00004770-200311000-00006

[iej14250-bib-0004] Bender, I.B. , Seltzer, S. & Soltanoff, W. (1966) Endodontic success—a reappraisal of criteria. Oral Surgery, Oral Medicine, and Oral Pathology, 22, 780–789. 5224186 10.1016/0030-4220(66)90368-9

[iej14250-bib-0005] Bornstein, M.M. , Lauber, R. , Sendi, P. & Von Arx, T. (2011) Comparison of periapical radiography and limited cone‐beam computed tomography in mandibular molars for analysis of anatomical landmarks before apical surgery. Journal of Endodontics, 37, 151–157. 21238794 10.1016/j.joen.2010.11.014

[iej14250-bib-0006] Bossuyt, P.M. , Reitsma, J.B. , Bruns, D.E. , Gatsonis, C.A. , Glasziou, P.P. , Irwig, L. et al. (2015) Stard 2015: an updated list of essential items for reporting diagnostic accuracy studies. BMJ [British Medical Journal], 351, h5527. 26511519 10.1136/bmj.h5527 PMC4623764

[iej14250-bib-0007] Cotton, T.P. , Geisler, T.M. , Holden, D.T. , Schwartz, S.A. & Schindler, W.G. (2007) Endodontic applications of cone‐beam volumetric tomography. Journal of Endodontics, 33, 1121–1132. 17931947 10.1016/j.joen.2007.06.011

[iej14250-bib-0008] Dutta, A. , Smith‐Jack, F. & Saunders, W.P. (2014) Prevalence of periradicular periodontitis in a Scottish subpopulation found on CBCT images. International Endodontic Journal, 47, 854–863. 24320040 10.1111/iej.12228

[iej14250-bib-0009] Dvoyris, V. (2023) Artificial intelligence as a day‐to‐day diagnostic aid in the dental practice. International Dentistry‐African Edition, 13, 20–25.

[iej14250-bib-0010] Faul, F. , Erdfelder, E. , Lang, A.G. & Buchner, A. (2007) G*power 3: a flexible statistical power analysis program for the social, behavioral, and biomedical sciences. Behavior Research Methods, 39, 175–191. 17695343 10.3758/bf03193146

[iej14250-bib-0011] Forsberg, J. & Halse, A. (1994) Radiographic simulation of a periapical lesion comparing the paralleling and the bisecting‐angle techniques. International Endodontic Journal, 27, 133–138. 7995645 10.1111/j.1365-2591.1994.tb00242.x

[iej14250-bib-0012] Goldman, M. , Pearson, A.H. & Darzenta, N. (1972) Endodontic success – who's reading the radiograph? Oral Surgery, Oral Medicine, and Oral Pathology, 33, 432–437. 4501172 10.1016/0030-4220(72)90473-2

[iej14250-bib-0013] Horner, K. , Eaton, K.A. , Royal College of Surgeons of England, F.O.G.D.P.S. & Practice, R.C.O.S.O.E.F.O.G.D. (2018) *Selection Criteria For Dental Radiography*, FGDP(UK).

[iej14250-bib-0014] Huumonen, S. & Ørstavik, D. (2002) Radiological aspects of apical periodontitis. Endodontic Topics, 1, 3–25.

[iej14250-bib-0015] Issa, J. , Jaber, M. , Rifai, I. , Mozdziak, P. , Kempisty, B. & Dyszkiewicz‐Konwińska, M. (2023) Diagnostic test accuracy of artificial intelligence in detecting periapical periodontitis on two‐dimensional radiographs: a retrospective study and literature review. Medicina, 59, 768. 37109726 10.3390/medicina59040768 PMC10142688

[iej14250-bib-0016] Kanagasingam, S. , Lim, C.X. , Yong, C.P. , Mannocci, F. & Patel, S. (2017) Diagnostic accuracy of periapical radiography and cone beam computed tomography in detecting apical periodontitis using histopathological findings as a reference standard. International Endodontic Journal, 50, 417–426. 27063209 10.1111/iej.12650

[iej14250-bib-0017] Khanagar, S.B. , Al‐Ehaideb, A. , Maganur, P.C. , Vishwanathaiah, S. , Patil, S. , Baeshen, H.A. et al. (2021) Developments, application, and performance of artificial intelligence in dentistry – a systematic review. Journal of Dental Sciences, 16, 508–522. 33384840 10.1016/j.jds.2020.06.019 PMC7770297

[iej14250-bib-0018] Knight, A. , Blewitt, I. , Al‐Nuaimi, N. , Watson, T. , Herzog, D. , Festy, F. et al. (2020) Rapid chairside microbial detection predicts endodontic treatment outcome. Journal of Clinical Medicine, 9, 2086. 32635158 10.3390/jcm9072086 PMC7408726

[iej14250-bib-0019] Kruse, C. , Spin‐Neto, R. , Evar Kraft, D.C. , Vaeth, M. & Kirkevang, L.L. (2019) Diagnostic accuracy of cone beam computed tomography used for assessment of apical periodontitis: an ex vivo histopathological study on human cadavers. International Endodontic Journal, 52, 439–450. 30267421 10.1111/iej.13020

[iej14250-bib-0020] Lofthag‐Hansen, S. , Huumonen, S. , Gröndahl, K. & Gröndahl, H.G. (2007) Limited cone‐beam CT and intraoral radiography for the diagnosis of periapical pathology. Oral Surgery, Oral Medicine, Oral Pathology, Oral Radiology, and Endodontics, 103, 114–119. 17178504 10.1016/j.tripleo.2006.01.001

[iej14250-bib-0021] Low, K.M. , Dula, K. , Bürgin, W. & Von Arx, T. (2008) Comparison of periapical radiography and limited cone‐beam tomography in posterior maxillary teeth referred for apical surgery. Journal of Endodontics, 34(5), 557–562. Available from: 10.1016/j.joen.2008.02.022 18436034

[iej14250-bib-0022] Meirinhos, J. , Martins, J.N.R. , Pereira, B. , Baruwa, A. , Gouveia, J. , Quaresma, S.A. et al. (2020) Prevalence of apical periodontitis and its association with previous root canal treatment, root canal filling length and type of coronal restoration – a cross‐sectional study. International Endodontic Journal, 53, 573–584. 31749154 10.1111/iej.13256

[iej14250-bib-0023] Nagendrababu, V. , Pigg, M. , Duncan, H.F. , Abbott, P.V. , Fouad, A.F. , Kruse, C. et al. (2024) Pridase 2024 guidelines for reporting diagnostic accuracy studies in endodontics: a consensus‐based development. International Endodontic Journal, 57, 996–1005. 38669132 10.1111/iej.14075

[iej14250-bib-0024] Nair, P.N. (2004) Pathogenesis of apical periodontitis and the causes of endodontic failures. Critical Reviews in Oral Biology and Medicine, 15(6), 348–381. Available from: 10.1177/154411130401500604 15574679

[iej14250-bib-0025] Patel, N. , Khan, I. , Jarad, F. , Zavattini, A. , Koller, G. , Pimentel, T. et al. (2025) The short‐term postoperative pain and impact upon quality of life of pulpotomy and root canal treatment, in teeth with symptoms of irreversible pulpitis: a randomized controlled clinical trial. International Endodontic Journal, 58, 55–70. 39325552 10.1111/iej.14144 PMC11629050

[iej14250-bib-0026] Patel, S. , Brown, J. , Pimentel, T. , Kelly, R.D. , Abella, F. & Durack, C. (2019) Cone beam computed tomography in endodontics – a review of the literature. International Endodontic Journal, 52, 1138–1152. 30868610 10.1111/iej.13115

[iej14250-bib-0027] Patel, S. , Dawood, A. , Whaites, E. & Pitt Ford, T. (2009) New dimensions in endodontic imaging: part 1. Conventional and alternative radiographic systems. International Endodontic Journal, 42, 447–462. 19298577 10.1111/j.1365-2591.2008.01530.x

[iej14250-bib-0028] Patel, S. , Wilson, R. , Dawood, A. & Mannocci, F. (2012) The detection of periapical pathosis using periapical radiography and cone beam computed tomography – part 1: pre‐operative status. International Endodontic Journal, 45, 702–710. 22188219 10.1111/j.1365-2591.2011.01989.x

[iej14250-bib-0029] Rahim, N. (2024) *The impact of preservation of tooth structure on the outcome of endodontic and restorative treatment*. PhD thesis, King's College London.

[iej14250-bib-0030] Sadr, S. , Mohammad‐Rahimi, H. , Motamedian, S.R. , Zahedrozegar, S. , Motie, P. , Vinayahalingam, S. et al. (2023) Deep learning for detection of periapical radiolucent lesions: a systematic review and meta‐analysis of diagnostic test accuracy. Journal of Endodontics, 49, 248–261 E3. 36563779 10.1016/j.joen.2022.12.007

[iej14250-bib-0031] Scarfe, W.C. & Farman, A.G. (2008) What is cone‐beam CT and how does it work? Dental Clinics of North America, 52(4), 707–730. Available from: 10.1016/j.cden.2008.05.005 18805225

[iej14250-bib-0032] Schwartz, S.F. & Foster, J.K., Jr. (1971) Roentgenographic interpretation of experimentally produced bony lesions. I. Oral Surgery, Oral Medicine, and Oral Pathology, 32, 606–612. 5285698 10.1016/0030-4220(71)90326-4

[iej14250-bib-0033] Tibúrcio‐Machado, C. , Michelon, C. , Zanatta, F. , Gomes, M.S. , Marin, J.A. & Bier, C.A. (2021) The global prevalence of apical periodontitis: a systematic review and meta‐analysis. International Endodontic Journal, 54, 712–735. 33378579 10.1111/iej.13467

[iej14250-bib-0034] Vande Voorde, H.E. & Bjorndahl, A.M. (1969) Estimating endodontic “working length” with paralleling radiographs. Oral Surgery, Oral Medicine, and Oral Pathology, 27, 106–110. 5249154 10.1016/0030-4220(69)90037-1

[iej14250-bib-0035] Webber, R.L. & Messura, J.K. (1999) An in vivo comparison of diagnostic information obtained from tuned‐aperture computed tomography and conventional dental radiographic imaging modalities. Oral Surgery, Oral Medicine, Oral Pathology, Oral Radiology, and Endodontics, 88, 239–247. 10468470 10.1016/s1079-2104(99)70122-8

[iej14250-bib-0036] Zahran, S. , Patel, S. , Koller, G. & Mannocci, F. (2021) The impact of an enhanced infection control protocol on molar root canal treatment outcome – a randomized clinical trial. International Endodontic Journal, 54, 1993–2005. 34352123 10.1111/iej.13605

