# Supplementary material for: The detection of apical radiolucencies in periapical radiographs: A comparison between an artificial intelligence platform and expert endodontists with CBCT serving as the diagnostic benchmark
Source: Int Endod J. 2025 May 3;58(8):1146–57. doi: 10.1111/iej.14250 (PMC12254525; doi:10.1111/iej.14250)
Supplement: Supplementary file 1 — Data S1 [file IEJ-58-1146-s001.docx]

**Supplementary Figure 1:** Receiver operating characteristic (ROC) curves comparing Diagnocat and clinician performance in detecting apical radiolucencies on periapical radiographs, using cone-beam computed tomography (CBCT) as the reference standard, at the (A) tooth level and (B) root level (Diagnocat at 30% and 50% Probability Thresholds).

| **(N, %)** | | **CBCT** | | **Clinician** | | **Diagnocat** | |
| --- | --- | --- | --- | --- | --- | --- | --- |
|  |  |  |  | **Positive** | **Negative** | **Positive** | **Negative** |
| **All teeth**  (339, 100%) | | **Positive** | 121 | 79 | 42 | 58 | 63 |
|  |  | **Negative** | 218 | 5 | 213 | 10 | 208 |
| **Arch** | Upper  (137, 40.4%) | **Positive** | 53 | 35 | 18 | 22 | 31 |
|  |  | **Negative** | 84 | 2 | 82 | 2 | 82 |
|  | Lower  (202, 59.6%) | **Positive** | 68 | 44 | 24 | 36 | 32 |
|  |  | **Negative** | 134 | 3 | 131 | 8 | 126 |
| **Tooth Types**^†^ | Anterior  (10, 2.9%) | **Positive** | 8 | 8 | 0 | 5 | 3 |
|  |  | **Negative** | 2 | 0 | 2 | 1 | 1 |
|  | Molar  (327, 96.5%) | **Positive** | 113 | 71 | 42 | 53 | 60 |
|  |  | **Negative** | 214 | 5 | 209 | 9 | 205 |
| **Teeth** | L6  (126, 37.2%) | **Positive** | 45 | 33 | 12 | 30 | 15 |
|  |  | **Negative** | 81 | 0 | 81 | 5 | 76 |
|  | L7  (70, 20.6%) | **Positive** | 19 | 7 | 12 | 4 | 15 |
|  |  | **Negative** | 51 | 3 | 48 | 2 | 49 |
|  | U6  (79, 23.3%) | **Positive** | 35 | 23 | 12 | 14 | 21 |
|  |  | **Negative** | 44 | 1 | 43 | 0 | 44 |
|  | U7  (51, 15.0%) | **Positive** | 14 | 8 | 6 | 5 | 9 |
|  |  | **Negative** | 37 | 1 | 36 | 2 | 35 |

**Supplementary Table 1**: Cross-Tabulation of Clinician and Diagnocat Detection of Periapical Radiolucencies at Tooth Level Compared with CBCT Reference Standard (50% Probability Threshold)

^†^ Teeth with <5 counts excluded.

^§^ Tooth notation: L6 = mandibular first molar; L7 = mandibular second molar; U6 = maxillary first molar; U7 = maxillary second molar.

| **(N, %)** | | **CBCT** | | **Clinician** | | **Diagnocat** | |
| --- | --- | --- | --- | --- | --- | --- | --- |
|  |  |  |  | **Positive** | **Negative** | **Positive** | **Negative** |
| **All roots**  (794, 100%) | | **Positive** | **238** | 133 | 105 | 94 | 144 |
|  |  | **Negative** | **556** | 16 | 540 | 20 | 536 |
| **Arch** | Upper  (394, 49.6%) | **Positive** | **127** | 64 | 63 | 40 | 87 |
|  |  | **Negative** | **267** | 9 | 258 | 8 | 259 |
|  | Lower  (400, 50.4%) | **Positive** | **111** | 69 | 42 | 54 | 57 |
|  |  | **Negative** | **289** | 7 | 282 | 12 | 277 |
| **Tooth Types**^†^ | Anterior  (10, 1.3%) | **Positive** | **8** | 8 | 0 | 5 | 3 |
|  |  | **Negative** | **2** | 0 | 2 | 1 | 1 |
|  | Molar  (782, 98.5%) | **Positive** | **230** | 125 | 105 | 89 | 141 |
|  |  | **Negative** | **552** | 16 | 536 | 19 | 533 |
| **Roots**^†^ | Distal  (196, 24.7%) | **Positive** | **53** | 29 | 24 | 25 | 28 |
|  |  | **Negative** | **143** | 3 | 140 | 5 | 138 |
|  | Distobuccal  (128, 16.1%) | **Positive** | **39** | 18 | 21 | 8 | 31 |
|  |  | **Negative** | **89** | 5 | 84 | 3 | 86 |
|  | Mesial  (196, 24.7%) | **Positive** | **51** | 34 | 17 | 26 | 25 |
|  |  | **Negative** | **145** | 4 | 141 | 6 | 139 |
|  | Mesiobuccal  (128, 16.1%) | **Positive** | **40** | 22 | 18 | 15 | 25 |
|  |  | **Negative** | **88** | 2 | 86 | 2 | 86 |
|  | Palatal  (129, 16.3%) | **Positive** | **42** | 19 | 23 | 13 | 29 |
|  |  | **Negative** | **87** | 2 | 85 | 3 | 84 |
|  | Single rooted  (14, 1.8%) | **Positive** | **10** | 9 | 1 | 6 | 4 |
|  |  | **Negative** | **4** | 0 | 4 | 1 | 3 |

**Supplementary Table 2**: Cross-Tabulation of Clinician and Diagnocat Detection of Periapical Radiolucencies at Root Level Compared with CBCT Reference Standard (50% Probability Threshold)

^†^ Roots with <5 counts excluded.

**Supplementary Table 3:** Comparison of Correct Periapical Radiolucencies Detected by Clinicians and Diagnocat at the Tooth Level (50% Probability Threshold)

| **(N, %)** | | **Clinician** | **Diagnocat** | |
| --- | --- | --- | --- | --- |
|  |  |  | **Correct** | **Incorrect** |
| **All teeth**  (339, 100%) | | **Correct** | 260 | 32 |
|  |  | **Incorrect** | 6 | 41 |
| **CBCT PARLs** | Present  (121,35.7%) | **Correct** | 57 | 22 |
|  |  | **Incorrect** | 1 | 41 |
|  | Absent  (218, 64.3%) | **Correct** | 203 | 10 |
|  |  | **Incorrect** | 5 | 0 |
| **Arch** | Upper  (137, 40.4%) | **Correct** | 102 | 15 |
|  |  | **Incorrect** | 2 | 18 |
|  | Lower  (202, 59.6%) | **Correct** | 158 | 17 |
|  |  | **Incorrect** | 4 | 23 |
| **Tooth Types**^†^ | Anterior  (10, 2.9%) | **Correct** | 6 | 4 |
|  |  | **Incorrect** | 0 | 0 |
|  | Molar  (327, 96.5%) | **Correct** | 252 | 28 |
|  |  | **Incorrect** | 6 | 41 |
| **Teeth**^†^ | L6  (126, 37.2%) | **Correct** | 105 | 9 |
|  |  | **Incorrect** | 1 | 11 |
|  | L7  (70, 20.6%) | **Correct** | 50 | 5 |
|  |  | **Incorrect** | 3 | 12 |
|  | U6  (79, 23.3%) | **Correct** | 57 | 9 |
|  |  | **Incorrect** | 1 | 12 |
|  | U7  (51, 15.0%) | **Correct** | 39 | 5 |
|  |  | **Incorrect** | 1 | 6 |

^†^ Teeth with <5 counts excluded.

^§^ Tooth notation: L6 = mandibular first molar; L7 = mandibular second molar; U6 = maxillary first molar; U7 = maxillary second molar.

**Supplementary Table 4:** Comparison of Correct Periapical Radiolucencies Detected by Clinicians and Diagnocat at the Root Level (50% Probability Threshold)

| **(N, %)** | | **Clinician** | **Diagnocat** | |
| --- | --- | --- | --- | --- |
|  |  |  | **Correct** | **Incorrect** |
| **All roots**  (794, 100%) | | **Correct** | 610 | 63 |
|  |  | **Incorrect** | 20 | 101 |
| **CBCT PARLs** | Present  (238, 29.0%) | **Correct** | 85 | 48 |
|  |  | **Incorrect** | 9 | 96 |
|  | Absent  (556, 70.0%) | **Correct** | 525 | 15 |
|  |  | **Incorrect** | 11 | 5 |
| **Arch** | Upper  (394, 49.6%) | **Correct** | 287 | 35 |
|  |  | **Incorrect** | 12 | 60 |
|  | Lower  (400, 50.4%) | **Correct** | 323 | 28 |
|  |  | **Incorrect** | 8 | 41 |
| **Tooth Types**^†^ | Anterior  (10, 1.3%) | **Correct** | 6 | 4 |
|  |  | **Incorrect** | 0 | 0 |
|  | Molar  (782, 98.5%) | **Correct** | 602 | 59 |
|  |  | **Incorrect** | 20 | 101 |
| **Roots**^†^ | Distal  (196, 24.7%) | **Correct** | 158 | 11 |
|  |  | **Incorrect** | 5 | 22 |
|  | Distobuccal  (128, 16.1%) | **Correct** | 90 | 12 |
|  |  | **Incorrect** | 4 | 22 |
|  | Mesial  (196, 24.7%) | **Correct** | 162 | 13 |
|  |  | **Incorrect** | 3 | 18 |
|  | Mesiobuccal  (128, 16.1%) | **Correct** | 97 | 11 |
|  |  | **Incorrect** | 4 | 16 |
|  | Palatal  (129, 16.3%) | **Correct** | 93 | 11 |
|  |  | **Incorrect** | 4 | 21 |
|  | Single rooted  (14, 1.8%) | **Correct** | 9 | 4 |
|  |  | **Incorrect** | 0 | 1 |

^†^ Roots with <5 counts excluded.

**Supplementary Table 5:** Cross-Tabulation of Clinician and Diagnocat Detection of Periapical Radiolucencies at Tooth Level Compared with CBCT Reference Standard (30% Probability Threshold for Diagnocat Detection)

| **(N, %)** | | **CBCT** | | **Clinician** | | **Diagnocat** | |
| --- | --- | --- | --- | --- | --- | --- | --- |
|  |  |  |  | **Positive** | **Negative** | **Positive** | **Negative** |
| **All teeth**  (339, 100%) | | **Positive** | 121 | 79 | 42 | 85 | 36 |
|  |  | **Negative** | 218 | 5 | 213 | 48 | 170 |
| **Arch** | Upper  (137, 40.4%) | **Positive** | 53 | 35 | 18 | 35 | 18 |
|  |  | **Negative** | 84 | 2 | 82 | 14 | 70 |
|  | Lower  (202, 59.6%) | **Positive** | 68 | 44 | 24 | 50 | 18 |
|  |  | **Negative** | 134 | 3 | 131 | 34 | 100 |
| **Tooth Types**^†^ | Anterior  (10, 2.9%) | **Positive** | 8 | 8 | 0 | 6 | 2 |
|  |  | **Negative** | 2 | 0 | 2 | 2 | 0 |
|  | Molar  (327, 96.5%) | **Positive** | 113 | 71 | 42 | 79 | 34 |
|  |  | **Negative** | 214 | 5 | 209 | 46 | 168 |
| **Teeth** | L6  (126, 37.2%) | **Positive** | 45 | 33 | 12 | 37 | 8 |
|  |  | **Negative** | 81 | 0 | 81 | 19 | 62 |
|  | L7  (70, 20.6%) | **Positive** | 19 | 7 | 12 | 11 | 8 |
|  |  | **Negative** | 51 | 3 | 48 | 13 | 38 |
|  | U6  (79, 23.3%) | **Positive** | 35 | 23 | 12 | 21 | 14 |
|  |  | **Negative** | 44 | 1 | 43 | 6 | 38 |
|  | U7  (51, 15.0%) | **Positive** | 14 | 8 | 6 | 10 | 4 |
|  |  | **Negative** | 37 | 1 | 36 | 7 | 30 |

^†^ Teeth with <5 counts excluded.

^§^ Tooth notation: L6 = mandibular first molar; L7 = mandibular second molar; U6 = maxillary first molar; U7 = maxillary second molar.

| **(N, %)** | | **CBCT** | | **Clinician** | | **Diagnocat** | |
| --- | --- | --- | --- | --- | --- | --- | --- |
|  |  |  |  | **Positive** | **Negative** | **Positive** | **Negative** |
| **All roots**  (794, 100%) | | **Positive** | **238** | 133 | 105 | 131 | 107 |
|  |  | **Negative** | **556** | 16 | 540 | 77 | 479 |
| **Arch** | Upper  (394, 49.6%) | **Positive** | **127** | 64 | 63 | 58 | 69 |
|  |  | **Negative** | **267** | 9 | 258 | 29 | 238 |
|  | Lower  (400, 50.4%) | **Positive** | **111** | 69 | 42 | 73 | 38 |
|  |  | **Negative** | **289** | 7 | 282 | 48 | 241 |
| **Tooth Types**^†^ | Anterior  (10, 1.3%) | **Positive** | **8** | 8 | 0 | 6 | 2 |
|  |  | **Negative** | **2** | 0 | 2 | 2 | 0 |
|  | Molar  (782, 98.5%) | **Positive** | **230** | 125 | 105 | 125 | 105 |
|  |  | **Negative** | **552** | 16 | 536 | 75 | 477 |
| **Roots**^†^ | Distal  (196, 24.7%) | **Positive** | **53** | 29 | 24 | 34 | 19 |
|  |  | **Negative** | **143** | 3 | 140 | 23 | 120 |
|  | Distobuccal  (128, 16.1%) | **Positive** | **39** | 18 | 21 | 13 | 26 |
|  |  | **Negative** | **89** | 5 | 84 | 11 | 78 |
|  | Mesial  (196, 24.7%) | **Positive** | **51** | 34 | 17 | 35 | 16 |
|  |  | **Negative** | **145** | 4 | 141 | 24 | 121 |
|  | Mesiobuccal  (128, 16.1%) | **Positive** | **40** | 22 | 18 | 19 | 21 |
|  |  | **Negative** | **88** | 2 | 86 | 7 | 81 |
|  | Palatal  (129, 16.3%) | **Positive** | **42** | 19 | 23 | 20 | 22 |
|  |  | **Negative** | **87** | 2 | 85 | 10 | 77 |
|  | Single rooted  (14, 1.8%) | **Positive** | **10** | 9 | 1 | 8 | 2 |
|  |  | **Negative** | **4** | 0 | 4 | 2 | 2 |

**Supplementary Table 6:** Cross-Tabulation of Clinician and Diagnocat Detection of Periapical Radiolucencies at Root Level Compared with CBCT Reference Standard (30% Probability Threshold for Diagnocat Detection)

^†^ Roots with <5 counts excluded.

| **(N, %)** | **Sensitivity** | | | | | **Specificity** | | | | **Accuracy** | | | |
| --- | --- | --- | --- | --- | --- | --- | --- | --- | --- | --- | --- | --- | --- |
|  | **Clinician**  **(95% CI)** | **Diagnocat**  **(95% CI)** | **p-value^‡^** | **Clinician**  **(95% CI)** | | | **Diagnocat**  **(95% CI)** | **p-value^‡^** | **Clinician**  **(95% CI)** | | **Diagnocat**  **(95% CI)** | **p-value^‡^** |  |
| **All teeth**  **(339, 100%)** | 65.3%  (56.81, 73.77) | 70.3%  (62.10, 78.39) | 0.286 | 97.7%  (95.72, 99.69) | | | 78.0%  (72.48, 83.48) | <0.001*** | 86.1%  (82.46, 89.81) | | 75.2%  (70.63, 79.82) | <0.001*** |  |
| **Arch** |  |  |  |  | | |  |  |  | |  |  |  |
| Upper  (137, 40.4%) | 66.0%  (53.29, 78.79) | 66.0%  (53.29, 78.79) | 1.0 | 97.6%  (94.36, 100) | | | 83.3%  (75.36, 91.30) | 0.002** | 85.4%  (79.49, 91.31) | | 76.6%  (69.56, 83.73) | 0.017* |  |
| Lower  (202, 59.6%) | 64.7%  (53.35, 76.06) | 73.53%  (63.04, 84.02) | 0.18 | 97.8%  (95.26, 100) | | | 74.6%  (67.26, 81.99) | <0.001*** | 86.6%  (81.94, 91.33) | | 74.3%  (68.23, 80.29) | <0.001*** |  |
| **Tooth Type**^†^ |  |  |  |  | | |  |  |  | |  |  |  |
| Anterior  (10, 2.9%) | 100%  (67.56, 100) | 75.0%  (37.50, 100) | 0.5 | 100%  (34.24, 100) | | | 0.0%  (0.00, 0.00) |  | 100%  (72.25, 100) | | 60.0%  (29.64, 90.36) |  |  |
| Molar  (327, 96.4%) | 62.8%  (53.92, 71.74) | 69.9%  (61.46, 78.37) | 0.115 | 97.7%  (95.64, 99.69) | | | 78.5%  (73.0, 84.01) | <0.001*** | 85.6%  (81.82, 89.43) | | 75.5%  (70.88, 80.19) | <0.001*** |  |
| **Teeth**^†^ |  |  |  |  | | |  |  |  | |  |  |  |
| L6  (126, 37.2%) | 73.3%  (60.41, 86.25) | 82.2%  (71.05, 93.39) | 0.125 | 100%  (100.0, 100) | | | 76.5%  (67.32, 85.77) | <0.001*** | 90.5%  (85.35, 95.6) | | 78.6%  (71.41, 85.74) | 0.003** |  |
| L7  (70, 20.6%) | 36.8%  (15.15, 58.53) | 57.9%  (35.69, 80.10) | 0.289 | 94.1%  (87.66, 100) | | | 74.5%  (62.55, 86.47) | 0.021* | 78.6%  (68.96, 88.18) | | 70.0%  (59.26, 80.74) | 0.307 |  |
| U6  (79, 23.3%) | 65.7%  (49.99, 81.44) | 60.0%  (43.77, 76.23) | 0.687 | 97.7%  (93.32, 100) | | | 86.36%  (76.22, 96.50) | 0.063 | 83.5%  (75.37, 91.72) | | 74.7%  (65.10, 84.27) | 0.065 |  |
| U7  (51, 15.0%) | 57.1%  (31.22, 83.07) | 71.4%  (47.76, 95.09) | 0.5 | | 97.3%  (92.07, 100) | | 81.1%  (68.46, 93.70) | 0.07 | 86.3%  (76.83, 95.72) | | 78.4%  (67.14, 89.72) | 0.344 |  |

**Supplementary Table 7**: Diagnostic Performance of Clinicians and Diagnocat in Detecting Apical Radiolucencies: Tooth-Level Analysis of Sensitivity, Specificity, and Accuracy by Dental Arch and Tooth Type (30% Probability Threshold for Diagnocat Detection)

^†^Teeth with <5 counts excluded.

^‡^McNemar test used for clinician- Diagnocat comparison. *p < 0.05, **p < 0.01, ***p < 0.001.

^§^ Tooth notation: L6 = mandibular first molar; L7 = mandibular second molar; U6 = maxillary first molar; U7 = maxillary second molar.

| **(N, %)** | **Sensitivity** | | | | **Specificity** | | | | **Accuracy** | | | |
| --- | --- | --- | --- | --- | --- | --- | --- | --- | --- | --- | --- | --- |
|  | **Clinician**  **(95% CI)** | **Diagnocat**  **(95% CI)** | **p-value^‡^** | **Clinician**  **(95% CI)** | | **Diagnocat**  **(95% CI)** | **p-value^‡^** | **Clinician**  **(95% CI)** | | **Diagnocat**  **(95% CI)** | **p-value^‡^** |  |
| **All roots**  (794, 100%) | 55.9%  (49.57, 62.19) | 55.0%  (48.72, 61.36) | 0.795 | 97.1%  (95.73, 98.51) | | 86.2%  (83.28, 89.02) | <0.001*** | 84.8%  (82.26, 87.26) | | 76.8%  (73.89, 79.76) | <0.001*** |  |
| **Arch** |  |  |  |  | |  |  |  | |  |  |  |
| Upper  (394, 49.6%) | 50.4%  (41.7, 59.09) | 45.7%  (37.01, 54.33) | 0.296 | 96.6%  (94.46, 98.79) | | 89.1%  (85.41, 92.87) | <0.001*** | 81.7%  (77.91, 85.54) | | 75.1%  (70.86, 79.40) | 0.001*** |  |
| Lower  (400, 50.4%) | 62.2%  (53.14, 71.18) | 65.8%  (59.94, 74.59) | 0.557 | 97.6%  (95.81, 99.35) | | 83.4%  (79.10, 87.68) | <0.001*** | 87.8%  (84.54, 90.96) | | 78.5%  (74.47, 82.53) | <0.001*** |  |
| **Tooth Type**^†^ |  |  |  |  | |  |  |  | |  |  |  |
| Anterior  (10, 1.3%) | 100%  (67.56, 100) | 75.0%  (37.50, 100) | 0.5 | 100%  (34.24, 100) | | 0.0%  (0.00, 0.00) |  | 100%  (72.25, 100) | | 60.0%  (29.64, 90.36) |  |  |
| Molar  (782, 98.5%) | 54.4%  (47.91, 60.79) | 54.4%  (47.91, 60.79) | 1.0 | 97.1%  (95.7, 98.5) | | 86.4%  (83.55, 89.27) | <0.001*** | 84.5%  (81.99, 87.06) | | 77.0%  (74.03, 79.93) | <0.001*** |  |
| **Root**^†^ |  |  |  |  | |  |  |  | |  |  |  |
| Distal  (196, 24.7%) | 54.7%  (41.32, 68.12) | 64.2%  (51.24, 77.06) | 0.267 | 97.9%  (95.55, 100) | | 83.9%  (77.89, 89.94) | <0.001*** | 86.2%  (81.4, 91.05) | | 77.9%  (72.83, 84.32) | 0.017* |  |
| Distobuccal  (128, 16.1%) | 46.2%  (30.51, 61.8) | 33.3%  (18.54, 48.13) | 0.109 | 94.4%  (89.6, 99.17) | | 87.6%  (80.80, 94.48) | 0.109 | 79.7%  (72.72, 86.66) | | 71.1%  (63.24, 78.95) | 0.019* |  |
| Mesial  (196, 24.7%) | 66.7%  (53.73, 79.6) | 68.6%  (55.89, 81.36) | 1.0 | 97.2%  (94.58, 99.91) | | 83.5%  (77.40, 89.50) | <0.001*** | 89.3%  (84.96, 93.62) | | 79.6%  (73.95, 85.23) | 0.002** |  |
| Mesiobuccal  (128, 16.1%) | 55.0%  (39.58, 70.42) | 47.5%  (32025, 62.98) | 0.508 | 97.7%  (94.61, 100) | | 92.0%  (86.39, 97.70) | 0.125 | 84.4%  (78.08, 90.67) | | 78.1%  (70.96, 85.29) | 0.077 |  |
| Palatal  (129, 16.3%) | 45.2%  (30.19, 60.29) | 47.6%  (32.51, 62.72) | 1.0 | 97.6%  (94.55, 100) | | 88.5%  (81.80, 95.21) | 0.021* | 80.6%  (73.8, 87.44) | | 75.2%  (67.74, 82.65) | 0.210 |  |
| Single rooted  (14, 1.8%) | 90.0%  (71.41, 100) | 80.0%  (55.21, 100.00) | 1.0 | 100.0%  (100.0, 100) | | 50.0%  (1.00, 98.99) | 0.5 | 92.9%  (79.37, 100) | | 71.4%  (47.76, 95.09) | 0.375 |  |

**Supplementary Table 8:** Diagnostic Performance of Clinicians and Diagnocat in Detecting Apical Radiolucencies: Root-Level Analysis of Sensitivity, Specificity, and Accuracy by Dental Arch, Tooth Type and Root Type (30% Probability Threshold for Diagnocat Detection)

^†^Roots with <5 counts excluded.

^‡^McNemar test used for clinician- Diagnocat comparison. *p < 0.05, **p < 0.01, ***p < 0.001.

| **(N, %)** | | **Clinician** | **Diagnocat** | |
| --- | --- | --- | --- | --- |
|  |  |  | **Correct** | **Incorrect** |
| **All teeth**  (339, 100%) | | **Correct** | 237 | 55 |
|  |  | **Incorrect** | 18 | 29 |
| **CBCT PARLs** | Present  (121,35.7%) | **Correct** | 71 | 8 |
|  |  | **Incorrect** | 14 | 28 |
|  | Absent  (218, 64.3%) | **Correct** | 166 | 47 |
|  |  | **Incorrect** | 4 | 1 |
| **Arch** | Upper  (137, 40.4%) | **Correct** | 100 | 17 |
|  |  | **Incorrect** | 5 | 15 |
|  | Lower  (202, 59.6%) | **Correct** | 137 | 38 |
|  |  | **Incorrect** | 13 | 14 |
| **Tooth Types**^†^ | Anterior  (10, 2.9%) | **Correct** | 6 | 4 |
|  |  | **Incorrect** | 0 | 0 |
|  | Molar  (327, 96.5%) | **Correct** | 229 | 51 |
|  |  | **Incorrect** | 18 | 29 |
| **Teeth**^†^ | L6  (126, 37.2%) | **Correct** | 95 | 19 |
|  |  | **Incorrect** | 4 | 8 |
|  | L7  (70, 20.6%) | **Correct** | 40 | 15 |
|  |  | **Incorrect** | 9 | 6 |
|  | U6  (79, 23.3%) | **Correct** | 57 | 9 |
|  |  | **Incorrect** | 2 | 11 |
|  | U7  (51, 15.0%) | **Correct** | 37 | 7 |
|  |  | **Incorrect** | 3 | 4 |

**Supplementary Table 9:** Comparison of Correct Periapical Radiolucencies Detected by Clinicians and DiagnocatI at the Tooth Level (30% Probability Threshold)

^†^ Teeth with <5 counts excluded.

^§^ Tooth notation: L6 = mandibular first molar; L7 = mandibular second molar; U6 = maxillary first molar; U7 = maxillary second molar.

| **(N, %)** | | **Clinician** | **Diagnocat** | |
| --- | --- | --- | --- | --- |
|  |  |  | **Correct** | **Incorrect** |
| **All roots**  (794, 100%) | | **Correct** | 574 | 99 |
|  |  | **Incorrect** | 36 | 85 |
| **CBCT PARLs** | Present  (238, 29.0%) | **Correct** | 103 | 30 |
|  |  | **Incorrect** | 28 | 77 |
|  | Absent  (556, 70.0%) | **Correct** | 471 | 69 |
|  |  | **Incorrect** | 8 | 8 |
| **Arch** | Upper  (394, 49.6%) | **Correct** | 279 | 43 |
|  |  | **Incorrect** | 17 | 55 |
|  | Lower  (400, 50.4%) | **Correct** | 295 | 56 |
|  |  | **Incorrect** | 19 | 30 |
| **Tooth Types**^†^ | Anterior  (10, 1.3%) | **Correct** | 6 | 4 |
|  |  | **Incorrect** | 0 | 0 |
|  | Molar  (782, 98.5%) | **Correct** | 566 | 95 |
|  |  | **Incorrect** | 36 | 85 |
| **Roots**^†^ | Distal  (196, 24.7%) | **Correct** | 144 | 25 |
|  |  | **Incorrect** | 10 | 17 |
|  | Distobuccal  (128, 16.1%) | **Correct** | 87 | 15 |
|  |  | **Incorrect** | 4 | 22 |
|  | Mesial  (196, 24.7%) | **Correct** | 148 | 27 |
|  |  | **Incorrect** | 8 | 13 |
|  | Mesiobuccal  (128, 16.1%) | **Correct** | 96 | 12 |
|  |  | **Incorrect** | 4 | 16 |
|  | Palatal  (129, 16.3%) | **Correct** | 89 | 15 |
|  |  | **Incorrect** | 8 | 17 |
|  | Single rooted  (14, 1.8%) | **Correct** | 9 | 4 |
|  |  | **Incorrect** | 1 | 0 |

**Supplementary Table 10:** Comparison of Correct Periapical Radiolucencies Detected by Clinicians and Diagnocat at the Root Level (30% Probability Threshold)

^†^ Roots with <5 counts excluded.
